# The Pentatricopeptide Repeat Protein MEF100 Is Required for the Editing of Four Mitochondrial Editing Sites in *Arabidopsis*

**DOI:** 10.3390/cells10020468

**Published:** 2021-02-22

**Authors:** Bernard Gutmann, Michael Millman, Lilian Vincis Pereira Sanglard, Ian Small, Catherine Colas des Francs-Small

**Affiliations:** Australian Research Council Centre of Excellence in Plant Energy Biology, School of Molecular Sciences, The University of Western Australia, Crawley, WA 6009, Australia; bernardgutmann67@gmail.com (B.G.); michael.r.c.millman@gmail.com (M.M.); lilianmaria.sanglard@curtin.edu.au (L.V.P.S.); ian.small@uwa.edu.au (I.S.)

**Keywords:** plant mitochondria, editing, pentatricopeptide repeat (PPR) proteins

## Abstract

In *Arabidopsis thaliana* there are more than 600 C-to-U RNA editing events in the mitochondria and at least 44 in the chloroplasts. Pentatricopeptide repeat (PPR) proteins provide the specificity for these reactions. They recognize RNA sequences in a partially predictable fashion via key amino acids at the fifth and last position in each PPR motif that bind to individual ribonucleotides. A combined approach of RNA-Seq, mutant complementation, electrophoresis of mitochondrial protein complexes and Western blotting allowed us to show that MEF100, a PPR protein identified in a genetic screen for mutants resistant to an inhibitor of γ -glutamylcysteine synthetase, is required for the editing of *nad1*-493, *nad4*-403, *nad7*-698 and *ccmF_N2_*-356 sites in Arabidopsis mitochondria. The absence of editing in *mef100* leads to a decrease in mitochondrial Complex I activity, which probably explains the physiological phenotype. Some plants have lost the requirement for MEF100 at one or more of these sites through mutations in the mitochondrial genome. We show that loss of the requirement for MEF100 editing leads to divergence in the MEF100 binding site.

## 1. Introduction

RNA editing is a post-transcriptional process that alters the nucleotide sequences of RNA molecules such that the information contained in the mature RNA differs from that defined in the genome [1]. In the mitochondria and chloroplasts of angiosperms, RNA editing occurs as singular cytidine (C) to uridine (U) changes by deamination [2]. Editing is catalysed by editosomes, multifactor protein-RNA complexes in which RNA recognition is mediated by pentatricopeptide repeat (PPR) proteins [3]. The majority of the PPR proteins involved in editing belong to the PLS subfamily whose PPR array is composed of three variants of PPR motifs named P, L and S [4]. The PLS proteins usually carry additional PPR-like motifs (E1 and E2) and a cytidine-deaminase-like domain (the DYW domain) at their C-terminus. The zinc-binding DYW domain is required for catalysis of the deamination reaction [5,6,7,8,9]. A PPR-RNA code was developed to describe the observed correlations between amino acids at two key positions of known editing factors and their aligned nucleotides on the transcripts to which they bind [1,10,11]. Subsequently, the amino acids at these positions were shown to bind to the aligned nucleotide via hydrogen bonds [12]. Although this code can be used to predict the targets of some PPR proteins [7,11,13,14,15,16], most PPR proteins required for the editing of mitochondrial sites in *Arabidopsis* have not been identified. The characterisation of new editing factors remains an important step in understanding how PPR proteins recognise their targets and the role of RNA editing in organelle gene expression.

In wild-type plants, exposure to buthionine sulfoximine (BSO) inhibits the activity of γ-glutamylcysteine synthetase, severely reducing the plant’s ability to produce the tripeptide glutathione (γ-L-glutamyl-L-cysteinylglycine, GSH) [17]. GSH is involved in multiple biochemical processes, including the detoxification of reactive oxygen species [18] and an inability to produce it results in a specific loss of root meristem activity, stopping root growth [19]. In a screen of Col-0 ecotype plants treated with ethane methyl sulfonate (EMS), 10 *bir* mutants (for BSO-insensitive roots), including two PPR mutants had almost normal root growth in the presence of BSO [20]. This BSO-insensitive-root phenotype observed in the *bir6* mutant [20] was also shared by the *Arabidopsis* Complex I mutants *css1* [21] and *otp439* [20,22], suggesting that the phenotype is related to the reduced activity of mitochondrial Complex I. In this paper, we show that the mutant named here as *mef100* (for *mitochondrial editing factor 100*) is unable to effectively edit four mitochondrial sites and has moderately reduced levels of assembled Complex I.

## 2. Materials and Methods

### 2.1. Plant Growth and Selection

Arabidopsis seeds were surface sterilized in a solution containing 70% (*v*/*v*) ethanol and 0.05% Triton X-100 for 5 min, washed in 100% ethanol and then dried in sterile conditions. The seeds were plated (0.5× MS medium), vernalized at 4 °C in the dark for at least 24 h and then grown under a long-day photoperiod (16 h light, 100 μmol photons m^−2^ s^−1^). Three weeks after plating, they were transferred to the soil.

The *mef100* mutant plants were obtained from Stanislav Kopriva’s lab at the Department of Metabolic Biology at the John Innes Centre (United Kingdom). Plants were confirmed for homozygosity for the point mutation in the coding sequence of the AT3G61170 gene by Sanger sequencing of the PCR product obtained using the primer pair Mef100_859F and Mef100_2211R (Table S1). The *mef100* mutants were complemented (see Section 2.2) by floral dip [23] and their seeds were screened for successful transformation on 0.5× MS media containing 25 μg/μL hygromycin. Complementation was confirmed by PCR using primer pair mef100_1865F and pGWB1_C-terminal_R-1 (Table S1).

### 2.2. Plant Complementation

The region of genomic DNA corresponding to the full *mef100* gene and 522 nt upstream of the *mef100* start codon was amplified by PCR from Col-0 genomic DNA using mef100_ups_attB_F and mef100-stop_attBR (Table S1), which was cloned into the Gateway donor vector pDONR207 (Thermo Fisher Scientific, Waltham, MA, USA) according to the recommended protocol and used to transform DH5α. The fragment verified by Sanger sequencing was cloned into the pGWB1 binary vector and transferred into plants via *Agrobacterium tumefaciens* transformation [23].

### 2.3. RNA Editing Analyses

RNA from young leaves of *mef100*, Columbia-0 and three complemented *mef100* lines was extracted and DNase treated as previously described [24]. Reverse transcription using random primers and SuperScript III (Thermo Fisher Scientific, Waltham, MA, USA) was done according to their recommended protocol. PCR of the cDNA of *mef100*, Columbia-0 and complemented lines was done using Taq polymerase (Thermo Fisher Scientific, Waltham, MA, USA) according to their recommended protocol using primers specific to each of the four editing sites. PCR of the *nad1*-493 site was done using the primer pair *nad1*-224-F and *nad1*-861-RV1; *nad4*-403 using *nad4*-176-F and *nad4*-830-RV1; *nad7*-698 using *nad7*-382-F and *nad7*-1004-RV1; *ccmF_N2_*-356 using *ccmF*_N2_-88-F and *ccmF*_N2_-500-RV1 (Table S1).

### 2.4. Analysis of RNA Editing in RNA-seq Data

Crude mitochondrial pellets were isolated from 8-week-old leaves of *mef100* and Col-0 as previously described [25]. Mitochondrial RNA was extracted from these crude mitochondrial fractions using TRIzol (Thermo Fisher Scientific, Waltham, MA, USA). Two μg of RNA were treated with TURBO DNase (Thermo Fisher Scientific, Waltham, MA, USA) for 1 h at 37 °C and tested for DNA contamination as previously described [26]. Three hundred µg of mitochondrial RNA were used for cDNA library preparation using the TruSeq Stranded mRNA Library Prep kit with RIBO zero (Illumina, San Diego, California). The sequencing run (50 nt, single read) was performed on an Illumina Hi-Seq 2500 platform. RNA editing analyses were carried out as described in [27]. Reads were analysed with the BBMap package (sourceforge.net/projects/bbmap/). Clumpify was used to remove optical duplicates (with parameters *dedupe optical dist = 40*), BBDuk was used to remove adapters (with parameters *ktrim = r k = 23 mink = 11 hdist = 1 tpe tbo ftm = 5*) and BBMap was used to map trimmed reads to the organelle genomes (with parameters *ambiguous = random*). Nucleotide counting to identify the extent of editing was carried out with Pyrimid (https://github.com/ian-small/pyrimid, accessed on 21 January 2021). Nucleotide count data was analysed statistically with a Fisher exact test as implemented in the Python scipy.stats package and the *p*-values were corrected for multiple testing using statsmodels.stats.multitest.multipletests with the Simes-Hochberg procedure. Odds ratios were calculated after adding a pseudocount of 0.5 to all observations to avoid division by zero.

### 2.5. Analysis of Complex I and Complex III

Crude membrane pellets were prepared from Arabidopsis leaves as previously described [25] and the complexes separated by BN-PAGE using precast NativePAGE^TM^ mini-gels (Thermo Fisher Scientific, Waltham, MA, USA). After the run, the gel was rinsed twice in H_2_O. To detect the NADH oxidase activity of Complex I, the gel was incubated in 50 mL of detection buffer (50 mg of β-Nicotinamide adenine dinucleotide, reduced disodium salt hydrate (NADH) and 50 mg of nitrotetrazolium blue chloride dissolved in 0.1 M Tris, pH 7.4) in the dark for 45 min. The reaction was stopped by incubating the gel in 5% acetic acid for 2 h.

Proteins separated by BN-PAGE were transferred onto a PVDF membrane (Bio-Rad, Hercules, CA, USA) for 1 h in cathode buffer [25] in the XCell tank (100 mA constant current). After transfer, the membrane was destained in 100% ethanol, washed and blocked for 1 h at RT in the Roche blocking reagent (Roche, Mannheim, Germany). Immunodetection was performed using anti-RISP antibody [28] at a 1/3000 dilution, followed by an anti-rabbit secondary antibody (Sigma Aldricht, St Louis, MI, USA) diluted 1/10,000 and subsequently revealed using Clarity ECL reagents (Bio-Rad, Hercules, CA, USA). For SDS PAGE and Western blotting, purified mitochondrial preparations from 4-week-old seedlings were used as previously described [29].

### 2.6. Prediction of Binding by Editing Factors

Known editing factors and their target sites were aligned (Table S2) and the frequencies with which each 5th/last amino acid combination in the PPR motifs aligned with A, C, G or U were calculated and converted to a scoring table by calculating the natural log of (10 + observed counts)/(10 + counts expected from overall amino acid frequencies). Scoring tables were calculated separately for each motif type (available at https://github.com/ian-small/PPRmatcher (accessed on 21 January 2021) in ‘scoring_tables/Millman’ and Table S3). Scores were then calculated for any PPR-RNA alignment by looking up the score for each motif-nucleotide alignment and summing them.

## 3. Results

### 3.1. Mef100 Carries a Mutation in AT3G61170

**Figure 1:**
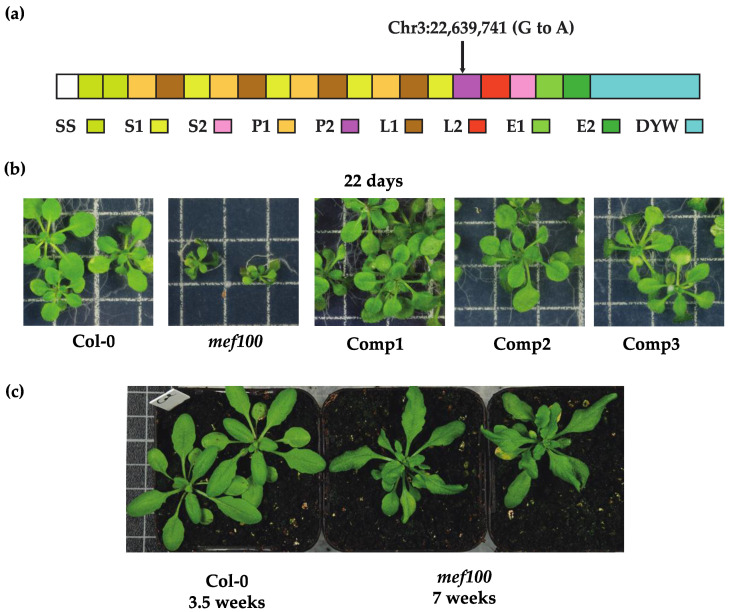
Characterisation of the *mef100* mutant. (**a**) Schematic representation of the architecture of the MEF100 protein (At3g61170). The EMS mutation creates a STOP codon in the P2 motif; (**b**) The *mef100* mutant shows delayed growth as compared with wild-type (Col-0) and 3 lines complemented with a functional copy of the *MEF100* gene (Comp1, 2 and 3); (**c**) *mef100* mutant plants sown 3.5 weeks before Col-0 plants reach comparable development stages.

The natural MEF100 protein consists of 17 PPR motifs and E1-E2-DYW C-terminal domains [4]. The *mef100* mutant previously isolated in a screen for BSO-insensitive *Arabidopsis* mutants [20] contains an EMS-induced point mutation in the gene AT3G61170 that converts the 1497th nucleotide in the coding sequence from a guanine into an adenosine, converting the 499th codon into a stop codon (Figure 1a). This mutation is predicted to terminate the translation of MEF100 in the first helix of the P2 motif (Figure 1a). When grown under standard long-day conditions, the *mef100* mutant shows a delayed growth rate compared to wild-type plants (Figure 1b). The wild-type phenotype is completely restored in complemented plants expressing a functional copy of the *MEF100* gene including 522 nucleotides upstream of the potential start codon (Figure 1b). Similar development stages are obtained in Col-0 grown for 3.5 weeks and in the mutant grown for 7 weeks (Figure 1c), but the mutant plants display a dark curly foliage typical for Complex I mutants [20,22].

### 3.2. MEF100 Is the Specificity Factor for Four Mitochondrial RNA Editing Sites

**Figure 2:**
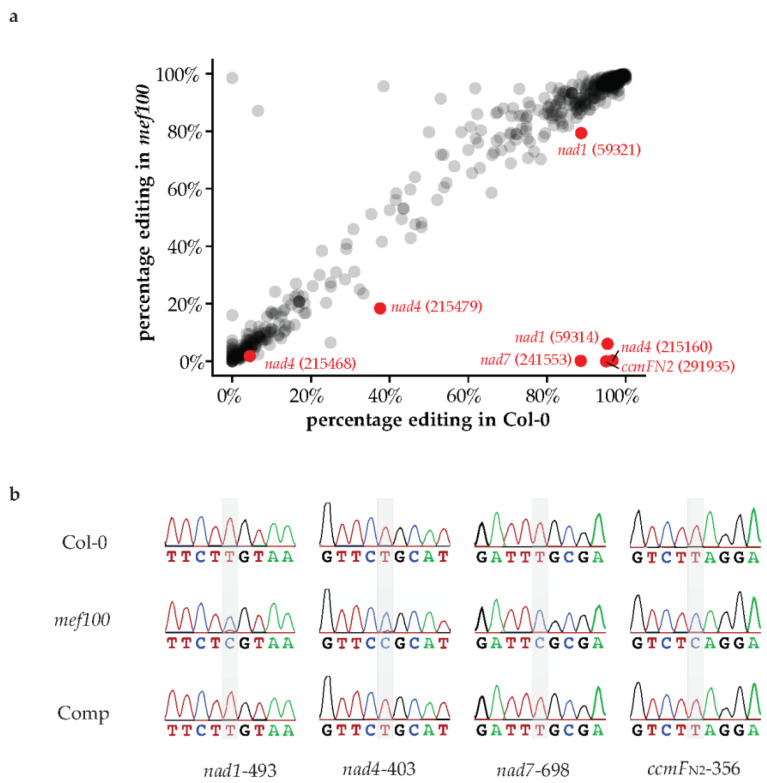
Analysis of RNA editing in the *mef100* mutant, the wild-type Col-0 and complemented line. (**a**) Dot plot of the difference in percentage of editing in the *mef100* mutant as compared with Col-0 at all positions found to show C/U variation in the mitochondrial transcriptome. The seven editing sites recognised by MEF100 are labelled in red. (**b**) Sanger sequencing of the RT-PCR products of the four major mitochondrial editing sites in *mef100*, Col-0 and complemented plants. The location of the MEF100 editing sites are highlighted in grey. Some of the sequences contain several editing sites and the sequences shown for Col-0 correspond to the fully edited versions.

Targeting prediction software predicts that MEF100 is targeted to mitochondria (TargetP: 0.935, Predotar: 0.48) [30,31]. The fact that *mef100* was isolated in a screen for mutants insensitive to BSO [20] suggests that MEF100, a DYW-PPR protein, could be acting in the mitochondrial compartment as an RNA-editing factor. Therefore, we searched for mitochondrial RNA editing events that showed severely reduced levels of editing in the *mef100* mutant by sequencing RNA from isolated mitochondria. Approximately 10 million reads were obtained by RNA-seq from each sample and mapped to the Arabidopsis organelle genomes. Of these, 1.01 M reads mapped to the mitochondrial genome in the wild-type sample and 8.97 M reads mapped to the mitochondrial genome in the *mef100* sample. The RNA nucleotides mapping to each position were counted to analyse RNA editing. Using a Fisher exact test, we identified seven sites that were differentially edited in the *mef100* mutant as compared with Col-0. Four sites showed dramatic decreases of editing in *mef100*: *nad1*-493 (genome position 59,314), *nad4*-403 (215,160), *nad7*-698 (241,553) and *ccmF_N2_*-356 (291,935), from 85–99% editing in Col-0 to 0% editing in the *mef100* mutant, except for the *nad1* site, which showed residual editing in *mef100* (Figure 2a,b and Table 1). The lack of editing at these sites in the *mef100* mutant strongly suggests that the MEF100 protein acts as the specificity factor for the editing reactions at these sites. Small but significant decreases were observed at three additional positions (*nad1*-500 (59,321), *nad4*-95 (215,468) and *nad4*-84 (215,479)), but we do not believe these sites are direct targets of MEF100 (see Section 4).

To confirm that the lack of a functional MEF100 protein was the cause of these editing deficiencies, the *mef100* mutant plants were complemented with a functional copy of the *MEF100* gene together with its native promoter. Sequencing of RT-PCR products from the complemented plants along with *mef100* mutant and Col-0 plants showed that wild-type editing levels were restored in the complemented plants at all four major editing sites. These results confirm that the MEF100 protein is required for these four editing events in wild-type Arabidopsis plants (Figure 2b).

### 3.3. Effect of MEF100-Mediated Editing Events at the Protein Level

All four of the main editing events that require MEF100 as the editing factor result in a codon that specifies a different amino acid from that of the unedited codon. Editing of *nad1*-493 and *nad4*-403 result in arginine (R) to cysteine (C) transitions while editing of *nad7*-698 and *ccmF_N2_*-356 result in serine (S) to leucine (L) transitions. The *nad1*, *nad4* and *nad7* transcripts all encode proteins that are subunits of mitochondrial Complex I (NADH-ubiquinone oxidoreductase), while the protein encoded by the *ccmF_N2_* transcript is a subunit of the cytochrome *c* maturation system [32] that attaches haem to apocytochromes. A comparison of the amino acid sequences of these four proteins revealed that the amino acids specified by these edited codons are conserved in other land plant species which lack these editing sites as well as in *Marchantia polymorpha*, a species devoid of RNA editing (Figure S1).

**Figure 3:**
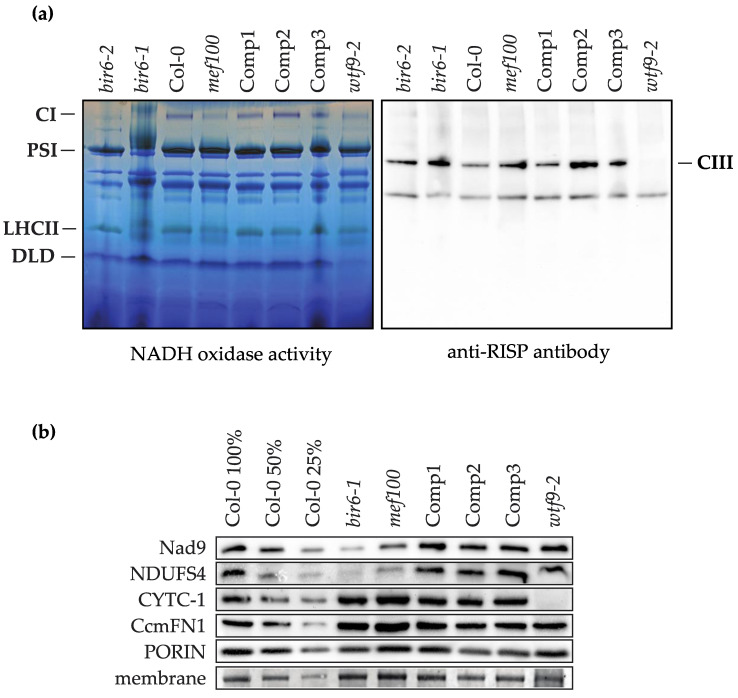
Analysis of mitochondrial respiratory complexes in *mef100* as compared with the *bir6-1* and *wtf9-2* mutants impaired in Complex I and III respectively, Col-0 and complemented plants (Comp1, 2, 3). (**a**) BN-PAGE analysis of total membrane extracts: Complex I (CI) and dihydrolipoamide dehydrogenase (DLD) activity bands are indicated on the BN gel (left panel) as well as photosystem I (PSI) and light harvesting complex II (LHCII). Complex III (CIII) is shown on a Western blot probed with an anti-RISP antibody. (**b**) Western blot analysis using antibodies against two subunits of Complex I (Nad9 and NDUFS4), CYTOCHROME *C1* (CYTC-1) and CcmF_N1_, a subunit of the CCM complex. The anti-porin antibody and a stained membrane were used as loading controls. The full images of the membranes are shown in Figure S2.

The fact that these amino acid changes are conserved in other species suggests that a lack of editing may impact the function of Complex I and/or the cytochrome *c* maturation complex in *Arabidopsis* mitochondria. Cytochrome *c_1_* within the cytochrome *bc_1_* complex (Complex III) requires haem attachment by the cytochrome *c* maturation complex for correct assembly and function [25], so to analyse the effect of the inactivation of MEF100 on the accumulation of complexes I and III, BN-PAGE was performed on leaf membrane proteins isolated from *mef100,* Col-0 and complemented *mef100* plants as well as the Complex I-deficient mutants *bir6*-1, *bir6*-2 [20] and the cytochrome *c* maturation (CCM)-deficient mutant *wtf9* [25]. In-gel NADH oxidase activity staining showed that the reduction in Complex I activity in *mef100*, less marked than that of *bir6-1* and *bir6-2*, was restored in *mef100* plants complemented with a functional *MEF100* gene (Figure 3a). This suggests that Complex I might be inefficiently assembled or unstable when it includes the three Nad subunits translated from unedited transcripts. This result is supported by the reduced levels of the two Complex I subunits Nad9 and NDUFS4 observed by Western blot (Figure 3b). In contrast, levels of Cytochrome *c1* and CcmF_N1_ were not affected in the *mef100* mutant and a Western blot of the BN gel probed with anti-RISP antibody confirmed that Complex III assembly was not affected in *mef100* (Figure 3a).

### 3.4. MEF100 Is a Good Match for Its Target According to the PPR Code

**Figure 4:**
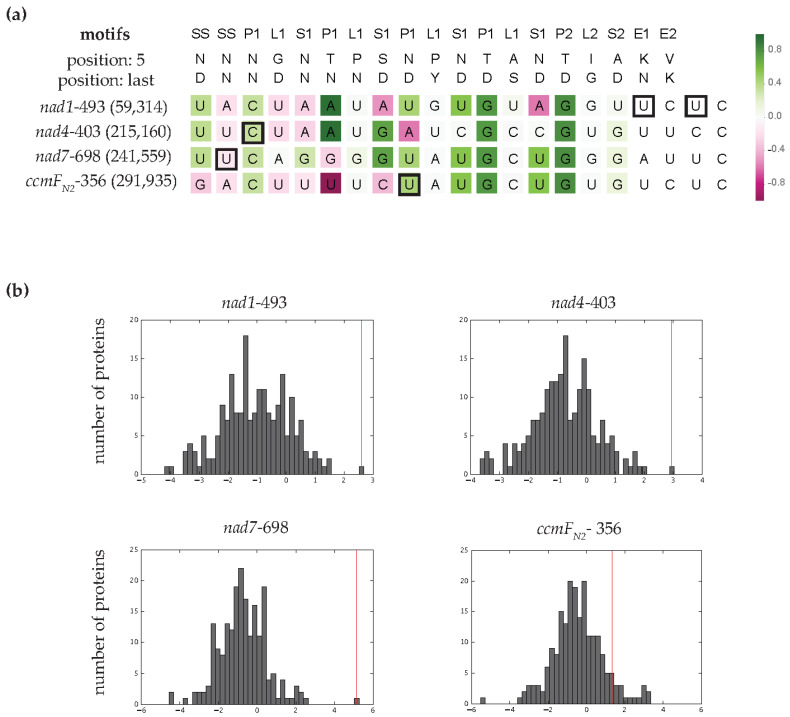
RNA target predictions for MEF100 according to the PPR code. (**a**) Scores of the MEF100 PPR motifs with the ribonucleotide sequences upstream of its target sites. The edited C is in last position in all four sequences. Motifs were aligned such that the E2 motif was aligned with the nucleotide two positions upstream of the edited C. Matches and mismatches of a PPR motif with the aligned nucleotide according to the PPR-RNA binding code are shown in green and magenta, respectively. The opacity is proportional to the strength of the correlation. The editing sites found upstream of the sites edited by MEF100 are indicated by a square. (**b**) Distribution of prediction scores for the MEF100 target sites aligned with 205 PLS-PPR proteins. Red lines indicate the prediction for the target sites aligned with MEF100.

PPR editing factors recognize their target sites via interactions between the 5th and last amino acids in each motif and the aligned RNA base [10,13]. These base preferences are to some extent predictable [7,11,13,14,15,16]. To assess whether we could have predicted MEF100 as the specificity factor for these four editing sites, we aligned them with the 205 potential PLS-PPR editing factors encoded by the *Arabidopsis* nuclear genome. The combined interactions of MEF100 PPR motifs with these target sequences produce very good scores according to the PPR-RNA binding code [10,13], with MEF100 being the top-ranked PLS-PPR editing factor for *nad1*-493, *nad4*-403 and *nad7*-698 (Figure 4b). In contrast, MEF100 is not the top predicted editing factor for the *ccmF_N2_*-356 site (Figure 4b), with 17 PLS-PPRs that score better predictions for the sequence.

We aligned MEF100 PPR motifs with the RNA sequences upstream of each edited cytidine (Figure 4a). Many of the predicted PPR-RNA interactions are in accordance with expectations from other editing factor interactions. In particular, five C-terminal motifs (P1–9, S1–11, P1–12, P2–15 and S2–17) match well to the *nad7*-698, *ccmF_N2_*-356, *nad1*-493 and/or *nad4*-403 sites. In these motifs, the amino acids likely to be involved in RNA recognition are highly conserved in putative MEF100 orthologues (Figure S1) with a few exceptions when one of the four editing sites is lost. A high proportion of the target nucleotides are conserved: seven out of eight nucleotides are identical between *nad1*-493 and *nad4*-403 editing sites at the 5′ end, and six out of seven nucleotides are identical between *nad7*-698 and *ccmF_N2_*-356 at the 3′ end of the putative MEF100 binding region. Within the Poaceae, the loss of the editing sites on *nad1* and *nad4* correlates with divergence or truncation of the N-terminus of the protein, e.g., eight N-terminal motifs are missing in *Zea mays* (Table S4). These observations suggest that the N-terminal PPR motifs are more important for *nad1* and *nad4* binding.

**Figure 5:**
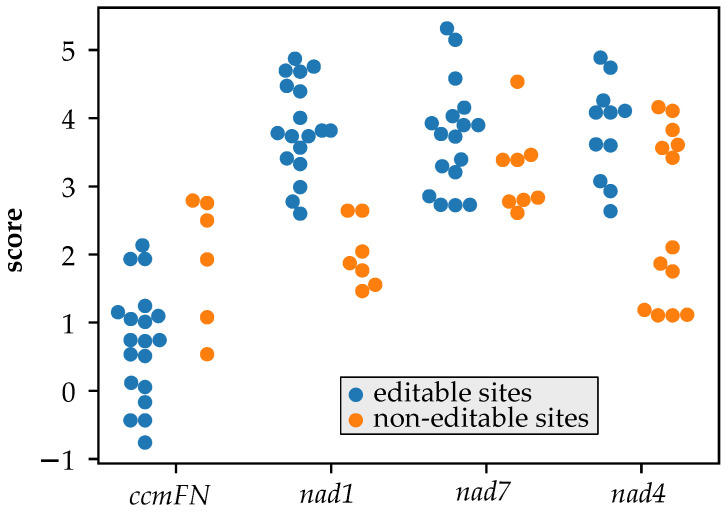
Variation in predicted recognition by MEF100 orthologues in different species. Putative MEF100 orthologues from a range of species where the mitochondrial genome has been sequenced were aligned to each of the 4 editing sites and the alignment scored using a scoring table developed from alignments of known editing factors and their targets. Higher scores indicate a better match between the protein and the RNA. For each site, species are assorted into two categories, those where the site is editable (C in the genome sequence; blue markers in the plot) and those where the site is not editable (T or rarely A or G in the genome sequence; orange markers in the plot). The non-editable sites score significantly lower for *nad1*-493 (*p*-value = 6 × 10^−7^) and *nad4*-403 (*p*-value = 0.003) but not for *nad7*-698 (*p*-value = 0.06) or *ccmF_N2_*-356 (*p*-value = 0.99) (one-sided *t*-test).

We scored how well different putative MEF100 orthologues were predicted to bind the four potential targets in their respective mitochondrial transcriptomes (Figure 5). For two sites (*nad1*-493 and *nad4*-403), the sequences scored significantly higher in species where the site is editable than in species where the site is not editable.

## 4. Discussion

We identified the PPR DYW protein MEF100 as involved in the editing of four mitochondrial sites. Three additional sites were flagged as significantly differentially edited between wild-type and the *mef100* mutant, but we think these are secondary effects and that these additional sites are not direct targets of MEF100. Firstly, these three sites are still edited at significant rates in *mef100*, which is not consistent with their editing being directly affected by MEF100. Secondly, all three sites are on the *nad1* or *nad4* transcripts and thus susceptible to be influenced by MEF100 binding or editing at the *nad1*-493 and *nad4*-403 sites that clearly are direct targets of MEF100. Editing at *nad1*-493 is seven nucleotides upstream of one of these 3 additional sites and thus presumably alters the cis-element required by the unknown factor that edits *nad1*-500. The two additional sites in *nad4* are ~300 nt upstream of the site at *nad4*-403, so the mechanism by which binding or editing of *nad4*-403 by MEF100 affects their editing is not so clear, but it may be by influencing RNA folding or turnover.

Complex I has an important function in cellular redox homeostasis, and when its activity is reduced, the alternative NADH oxidizing enzymes are induced. We previously showed that mutants with a reduced Complex I activity (*bir6*, *css1* and *otp439* mutants) had a reduced sensitivity to BSO-induced inhibition of root growth, because they retained more GSH and had a better ability to detoxify reactive oxygen species than wild-type plants [20]. RNA editing of the *nad1*-493, *nad4*-403 and *nad7*-698 sites has effects on the amino acid sequences of Complex I subunits. The accumulation of Complex I is disrupted in *mef100*, although not to the same extent as in the *bir6* mutant. The amino acid differences due to editing may play an important role in the interactions between Complex I subunits in the wild-type (WT) and thus the assembly or stability of the complex. We used the TMHMM software [33] to predict the transmembrane domains of wild-type Nad1 and Nad4 (components of the membrane arm of the L-shaped Complex I) and their counterparts in the *mef100* mutant. Although the predicted topology of the *mef100* form of Nad1 did not seem to be altered apart from minor changes in the fourth transmembrane domain (Figure S3), the modelling suggested quite major changes in the fourth transmembrane domain of Nad4 (Figure S4). These modifications could be sufficient to alter interactions between subunits and therefore the assembly of the complex. 

Nevertheless, the residual accumulation of Complex I may explain the mild growth phenotype of *mef100* as compared with the *tang2* [22] or *ndufs4* [34] mutants, which almost entirely lack assembled Complex I. Whilst editing at the *ccmF*_N2_-356 site would be expected to be important based on conservation of the amino acid specified when the site is edited, the absence of editing does not seem to have any noticeable impact on the function of the CcmF_N2_ protein. The accumulation of Complex III is not disrupted in *mef100*, and the levels of cytochrome *c1* are unchanged, suggesting that the CCM complex is functional in *mef100*. The topology of the *E. coli* CcmF integral membrane protein [35] shows that the leucine residue resulting from the *ccmF_N2_*-356 editing event is located in a trans-membrane alpha helix and the alignment of *Arabidopsis thaliana* CcmF_N1_ and CcmF_N2_ with *E. coli* and other plant CcmF proteins suggest that it is conserved (after considering RNA editing in plants). Although, unlike leucine, serine is not a hydrophobic amino acid, it is found in several places in trans-membrane helices of the bacterial CcmF and can provide the necessary H bonds to maintain the alpha helix structure. Our assumption is that the presence of serine rather than leucine at this position does not destabilise CcmF_N2_ or compromise the function of the CCM complex, at least under standard growth conditions. Interestingly, the lack of editing of *ccmF*_N_-1553 in *Zea mays*, which leads to a phenylalanine to serine substitution in a neighbouring region of the same protein, leads to a reduction in complex III, mitochondrial malfunction and an embryo-lethal phenotype [36]. In contrast to the leucine residue inserted as a result of *ccmF_N2_*-356 editing, which merely contributes to the secondary structure of a membrane helix, this phenylalanine residue is one of 3 highly conserved amino acids (**S**V**H**A**F** in *E. coli*) located in a loop where the histidine residue is crucial for haem attachment. 

The PPR-RNA interactions of MEF100 with its target sites are mostly consistent with what would be expected with the current understanding of the PPR-RNA binding code. When aligned with the sequences upstream of the *nad1*-493, *nad4*-403 and *nad7*-698 editing sites, the interactions between the majority of the PPR motifs of MEF100 and the corresponding ribonucleotides fit well with expectations from the analysis of other editing factors aligned to their targets [1,10,11,13]. In comparison to the other sites, the *ccmF_N2_*-356 binding sequence is a poorer match, consistent with it being a less important event. The loss of one or more of these target sites in different plants (via mutation of the genome sequence, usually to T, thus obviating the need for RNA editing at that site) allowed us to test the hypothesis that the sequence upstream of the editing site is constrained not only by the requirement to encode suitable amino acids but also by the requirement to bind MEF100. In plants that no longer edit a particular site, we thus hypothesised that the sequence would tend to diverge in a way that would not necessarily preserve the MEF100 binding site. For the *nad1*-493 and *nad4*-403 sites the results are consistent with this hypothesis, but for the *nad7*-698 and *ccmF_N2_*-356 sites we do not see a significant drop in the match score to MEF100 in species where these sites are no longer edited (Figure 5). This may indicate that the *nad1*-493 and *nad4*-403 sites are under the strongest selection for recognition by MEF100, and illustrates the potential complexity of the co-evolution of transcripts and editing factors when multiple sites are involved [37], which is frequently the case for mitochondrial editing factors.

## Figures and Tables

**Table 1:** Number of edited and unedited reads in the mitochondrial genome at the seven positions impacted in *mef100* with percentage editing in *mef100* and Col-0. The *p*-values for the Fisher exact test are given.

		Col-0			*mef100*			
Position	Annotation	Edited	Total	% Editing	Edited	Total	% Editing	*p*-Value
59314	*nad1* CGT(R) to TGT(C)	413	433	95.38%	381	6304	6.04%	0
59321	*nad1* TCG(S) to TTG(L)	1002	1130	88.67%	13358	16,837	79.34%	3.02 × 10^−13^
291935	*ccmFN_2_* TCA(S) to TTA(L)	38	40	95.00%	0	712	0.00%	3.20 × 10^−59^
215160	*nad4* CGC(R) to TGC(C)	291	301	96.68%	13	5429	0.24%	0
215468	*nad4* TCA(S) to TTA(L)	32	716	4.47%	254	14,186	1.79%	4.57 × 10^−3^
215479	*nad4* synonymous	197	524	37.60%	1606	8731	18.39%	1.13 × 10^−20^
241553	*nad7* TCG(S) to TTG(L)	813	918	88.56%	20	15,966	0.13%	0

## Data Availability

The RNA-seq data is available from SRA under the BioProject ID: PRJNA686212.

## Supplementary Materials

The following are available online at https://www.mdpi.com/2073-4409/10/2/468/s1, Figure S1: Conservation of MEF100 binding sites in land plants, Figure S2: Western blot membranes used in Figure 3, Figure S3: Trans-membrane helix predictions obtained by TMHMM for Nad1, Figure S4: Trans-membrane helix predictions obtained by TMHMM for Nad4, Table S1: Primers used in this work, Table S2: Alignment of known editing factors and their target sites, Table S3: Scoring tables, Table S4: Conservation of the amino acid 5 and last in MEF100 orthologs and the editing sites recognised by MEF100 in Arabidopsis.
