# Supplementary material for: The Pentatricopeptide Repeat Protein MEF100 Is Required for the Editing of Four Mitochondrial Editing Sites in Arabidopsis
Source: Cells. 2021 Feb 22;10(2):468. doi: 10.3390/cells10020468 (PMC7926422; doi:10.3390/cells10020468)
Supplement: Supplementary file 1 [file cells-10-00468-s001.zip › cells-1079802-SI-layout/Figure S1.pdf]

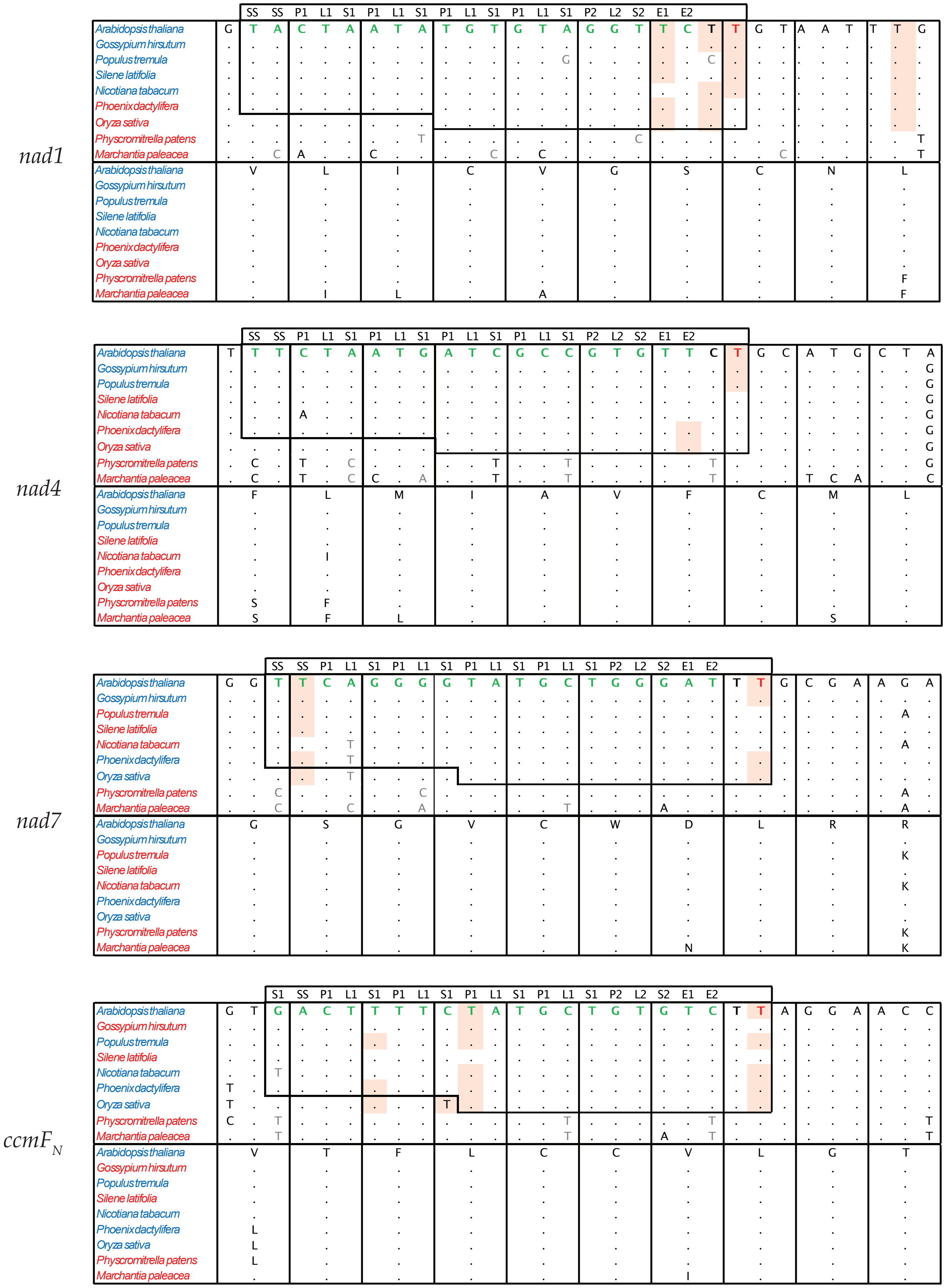

Figure S1 . Conservation of MEF100 binding sites in land plants**.** For each affected transcript, the nucleotide (top panel) and protein (bottom panel) sequences coded by the mature RNA from 5 dicotyledonous, two monocotyledonous, a moss and a liverwort species representing the diversity of binding site sequences and length of MEF100 observed in our dataset are compared with Arabidopsis (the dots correspond to a conservation of the nucleotide or amino acid). Editing sites are highlighted in orange. The names of the species editing the targets are in blue characters whilst the names of the species that lost the editing sites are in red. The MEF100 motifs and their targets are in black boxes.
