# Supplementary material for: The Pentatricopeptide Repeat Protein MEF100 Is Required for the Editing of Four Mitochondrial Editing Sites in Arabidopsis
Source: Cells. 2021 Feb 22;10(2):468. doi: 10.3390/cells10020468 (PMC7926422; doi:10.3390/cells10020468)
Supplement: Supplementary file 1 [file cells-10-00468-s001.zip › cells-1079802-SI-layout/Figure S2.pdf]

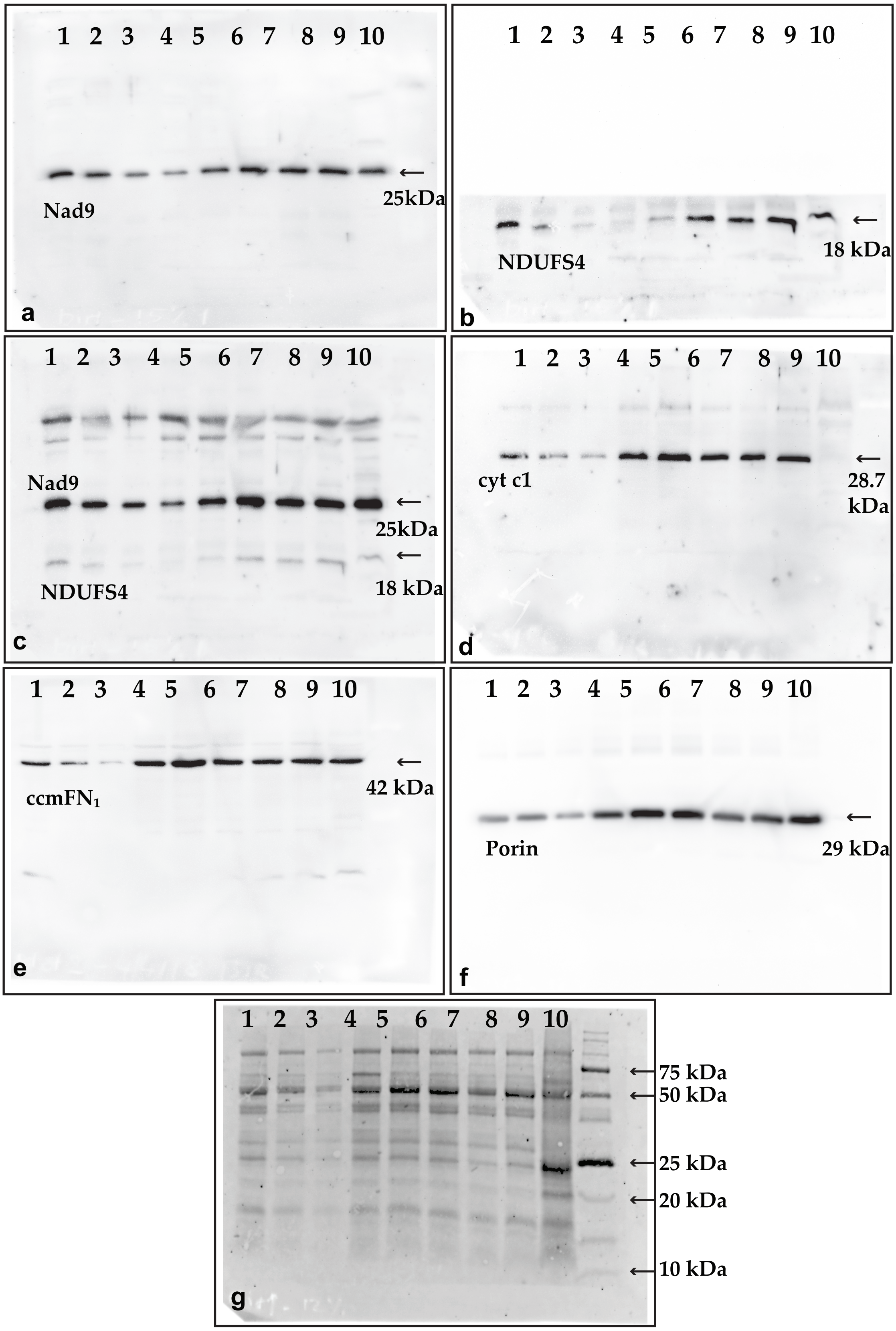

Supplementary Figure 2. Western blot membranes used in Figure 3. The names of the antibodies and the sizes of the signals are indicated on each membrane. The membrane shown in (a, b and c) was sequentially probed with antibodies recognising Nad9 (a) and NDUFS4 (b), and the Nad9 signal had to be masked during exposure to allow correct exposure of NDUFS4 (c). Lanes are 1: Col-0 100 %, 2: Col-0 50 %, 3: Col-0 25 %, 4: *bir6-1*, 5: *mef100*, 6: complemented line#1, 7: complemented line #2, 8: complemented line#3, 9: *wtf9-1*, 10: molecular weight markers.
