# Supplementary material for: The Pentatricopeptide Repeat Protein MEF100 Is Required for the Editing of Four Mitochondrial Editing Sites in Arabidopsis
Source: Cells. 2021 Feb 22;10(2):468. doi: 10.3390/cells10020468 (PMC7926422; doi:10.3390/cells10020468)
Supplement: Supplementary file 1 [file cells-10-00468-s001.zip › cells-1079802-SI-layout/Figure S3.pdf]

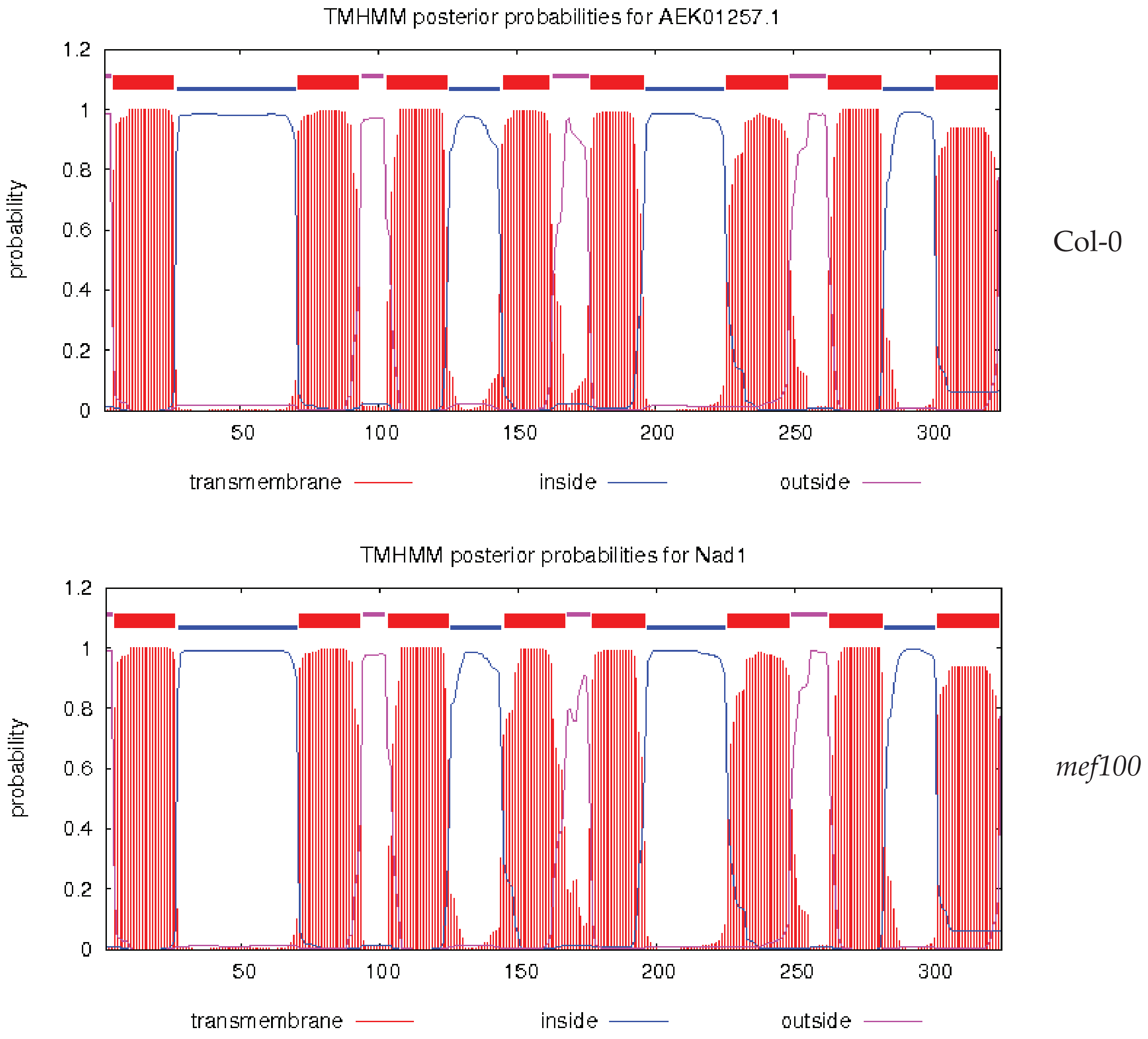

Figure S3. Trans-membrane helix predictions obtained by TMHMM for the WT Nad1 in Col-0 (top panel) and Nad1 in the *mef100* mutant (R165C, bottom panel). The trans-membrane segments are in thick red lines, the inside loops are represented by thin blue lines and the outside loops in thin magenta lines.
